# Effectiveness of psychosocial interventions on resilience, posttraumatic growth, and meaning in cancer survivors: a systematic review and meta-analysis of randomized controlled trials

**DOI:** 10.1192/j.eurpsy.2025.747

**Published:** 2025-08-26

**Authors:** G. Yıldız Aytaç, D. Hiçdurmaz, S. Karahan

**Affiliations:** 1 Psychiatric Nursing Department, Hacettepe University Faculty of Nursing; 2 Department of Biostatistics, Hacettepe University Faculty of Medicine, Ankara, Türkiye

## Abstract

**Introduction:**

A growing body of literature focuses on psychosocial interventions that include positive outcomes such as resilience, posttraumatic growth, and meaning in cancer survivors. A research synthesis is needed to provide a comprehensive understanding of this field.

**Objectives:**

This systematic review and meta-analysis of randomized controlled trials (RCTs) aimed to determine the effectiveness of psychosocial interventions on resilience, posttraumatic growth, and meaning in cancer survivors.

**Methods:**

CINAHL Plus with Full Text (EBSCOhost), CENTRAL, Pubmed, and WOS Core Collection databases were searched with no publication date or language restrictions. In addition, backward and forward citation searching was conducted in the Scopus database. The risk of bias of included studies was performed using the Revised Cochrane risk-of-bias tool for randomized trials (ROB2). The Grading of Recommendations, Assessment, Development, and Evaluation (GRADE) approach was used for rating the certainty of evidence assessment. Analyses were carried out using IBM SPSS Statistics 28, trial version.

**Results:**

The review included 14 RCTs with 1801 participants, published between 2005 and 2022. 10 of 14 RCTs reporting posttraumatic growth and/or meaning outcomes were involved in quantitative analysis. The overall risk of bias was judged as “some concerns” or “high” for all but one study. The pooled results suggested a small, beneficial effect in favor of psychosocial interventions on meaning “from pre-to immediately post-intervention,” compared with routine care (Cohen’s d = -0,298, 95% CI: -0,518 to -0,077; p = 0.008; I^2^ = 0%; 319 participants; low-certainty evidence), but no sustained effects “from pre-intervention to the longest follow-up” period (Cohen’s d = -0,172, 95% CI: -0,361 to 0,018; p = 0.075; I^2^ = 0%; 433 participants; low-certainty evidence). No significant differences were found for posttraumatic growth between the effects of psychosocial interventions and routine care “from pre-to immediately post-intervention” (Cohen’s d = -2.310, 95% CI: -5.735 to 1.115; p = 0.186; I^2^ = 99.6%; 498 participants; very low-certainty evidence) and “from pre-intervention to the longest follow-up” (Cohen’s d = -1.612, 95% CI: -3.847 to 0.623; p = 0.157; I^2^ = 99.6%; 947 participants; very low-certainty evidence) periods. The effects of psychosocial interventions on resilience could not be pooled since only two studies existed.

**Conclusions:**

The effectiveness of psychosocial interventions on all study outcomes is uncertain, and additional evidence are needed. Future RCTs of high methodological quality are warranted to meet the robust effects of psychosocial interventions in cancer survivors.

**Disclosure of Interest:**

None Declared

